# Supplementary material for: Construction and Preclinical Evaluation of a Recombinant Attenuated Measles Vaccine Candidate of the H1a Genotype
Source: Vaccines (Basel). 2025 May 27;13(6):571. doi: 10.3390/vaccines13060571 (PMC12197764; doi:10.3390/vaccines13060571)
Supplement: Supplementary file 1 [file vaccines-13-00571-s001.zip › vaccines-3627954-supplementary.pdf]

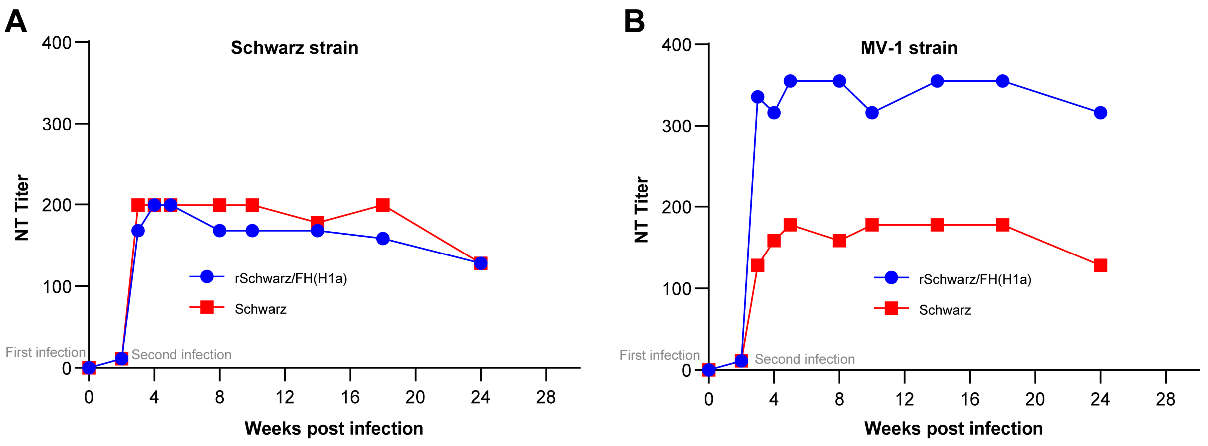

Figure S1. rSchwarz/FH(H1a) Exhibits Good immune persistence.

Each five female BALB/c mice, aged at 6-7 weeks and weighed between 19-21g, were intraperitoneally immunized with either the rSchwarz or rSchwarz/FH (H1a) viruses with 1 × 10^5 $CCID_{50}$ on day 0 and a second immunization on day14. 0,7,21,56,64,84,112 and 154 days after the second immunization, serum samples were collected from the mice to determine the neutralizing antibody titers against the Schwarz**(A)** and MV-1 viruses**(B)**.

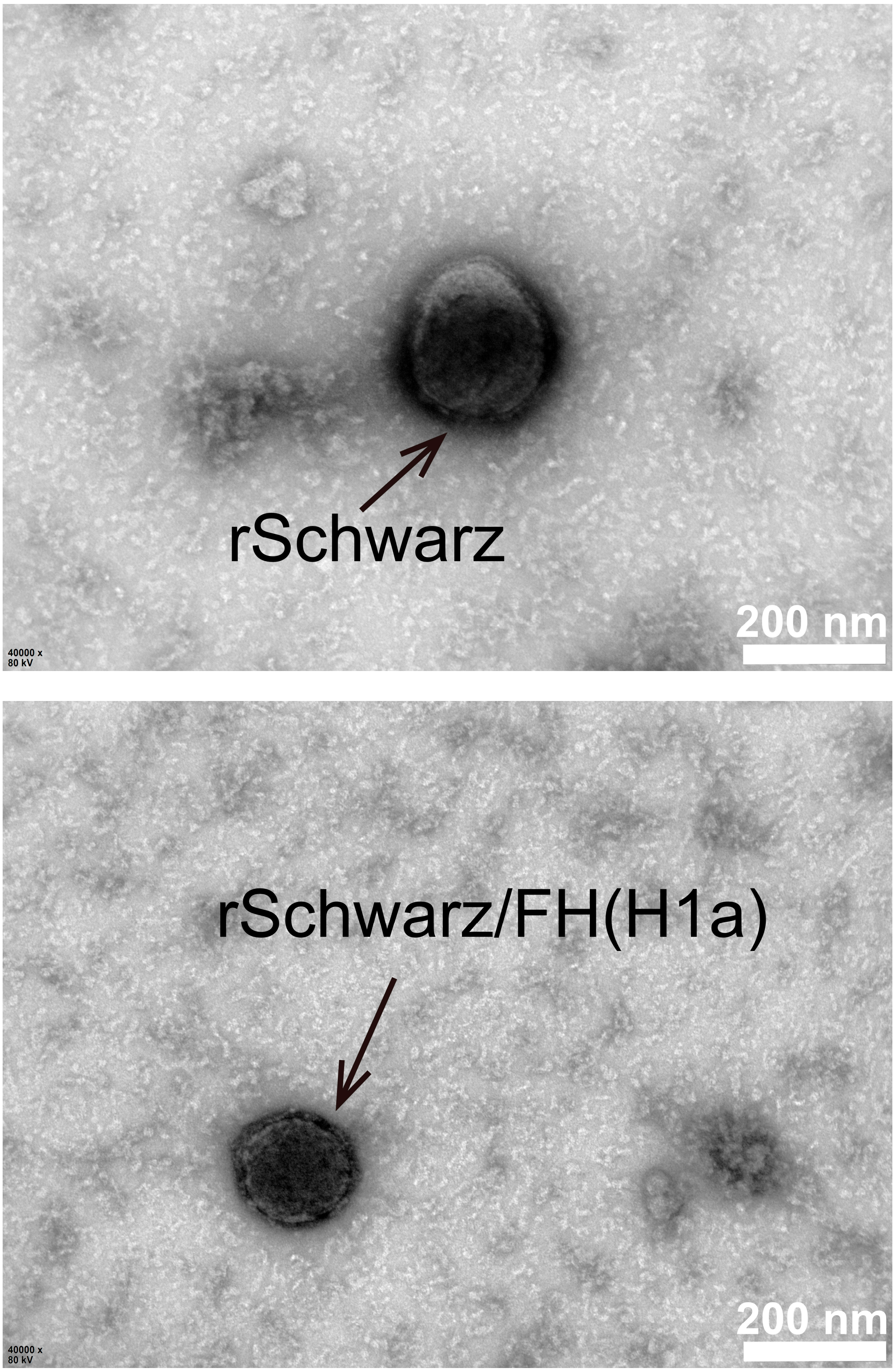

Figure S2. Electron Microscopic Observations of rSchwarz and rSchwarz/FH(H1a).

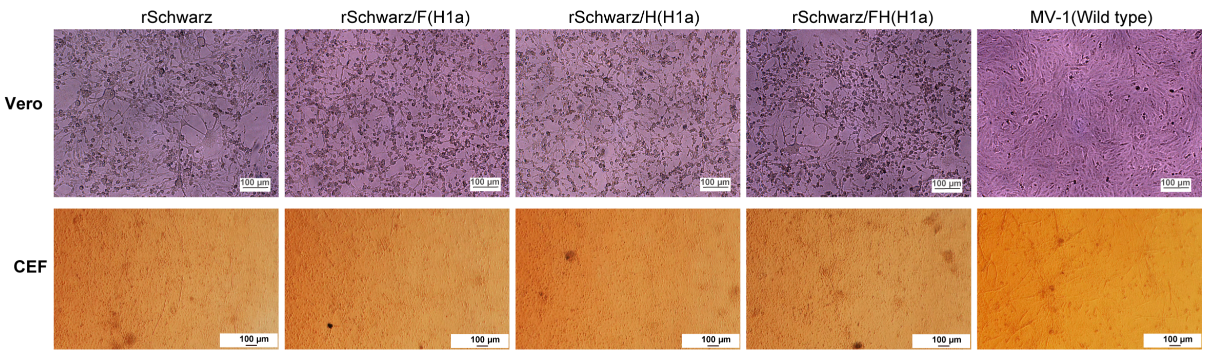

Figure S3. Cytopathic Effect Observation in Vero and CEF Cell Lines Infected with Measles Virus Recombinants.
